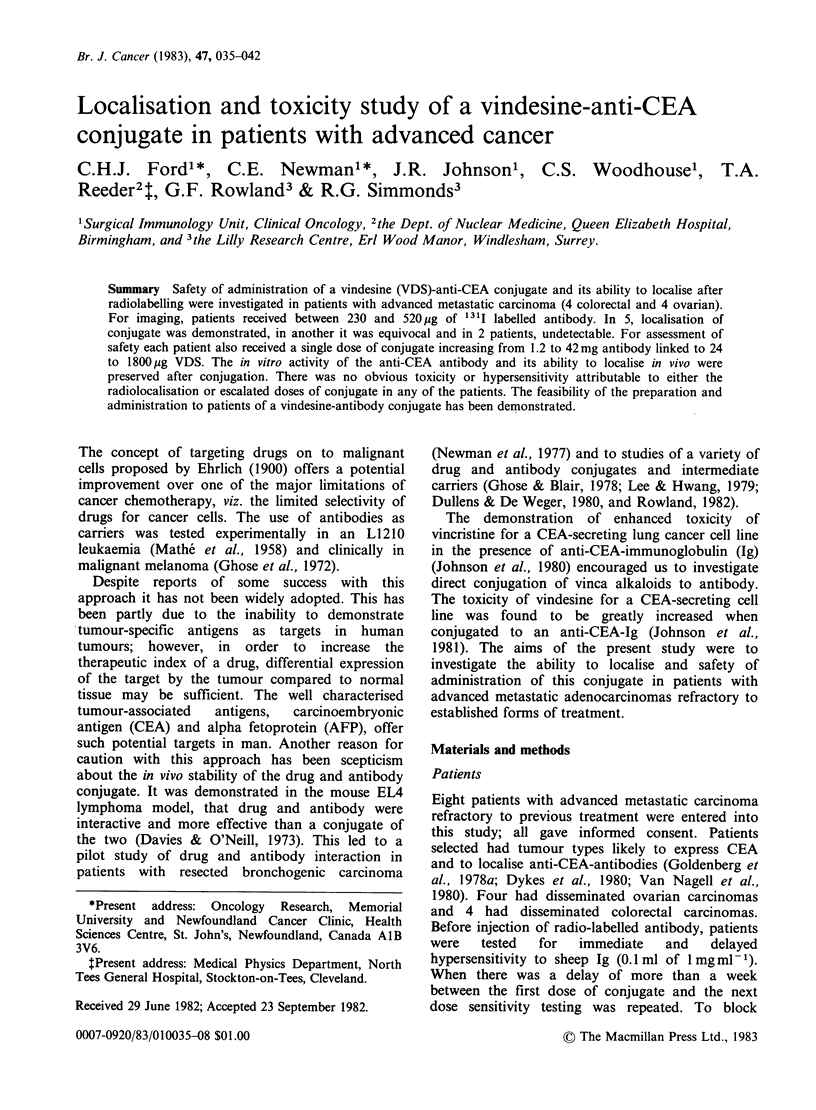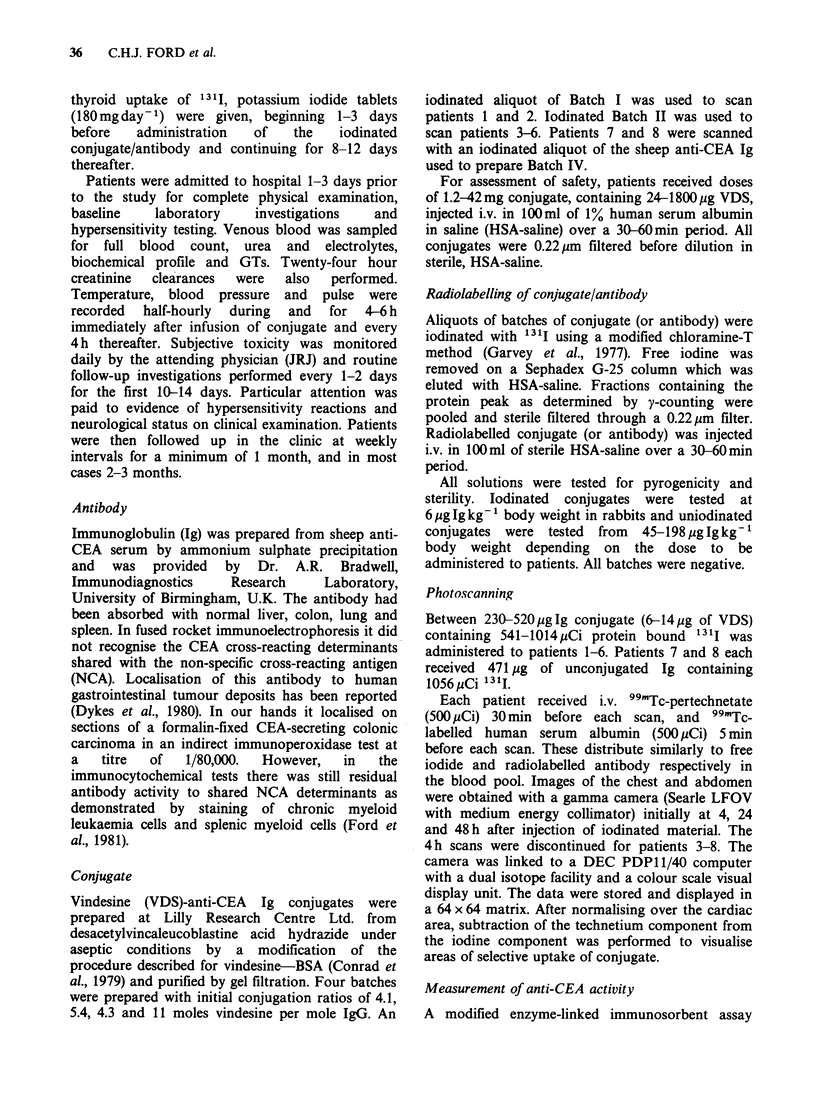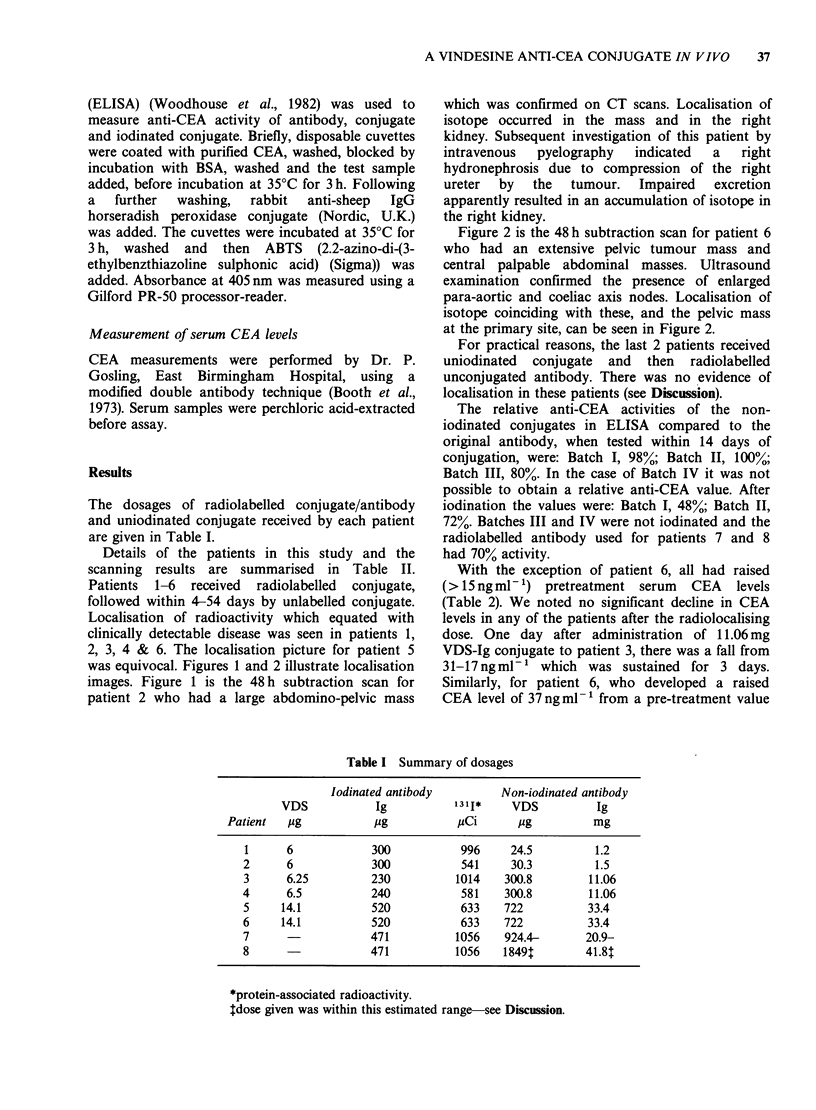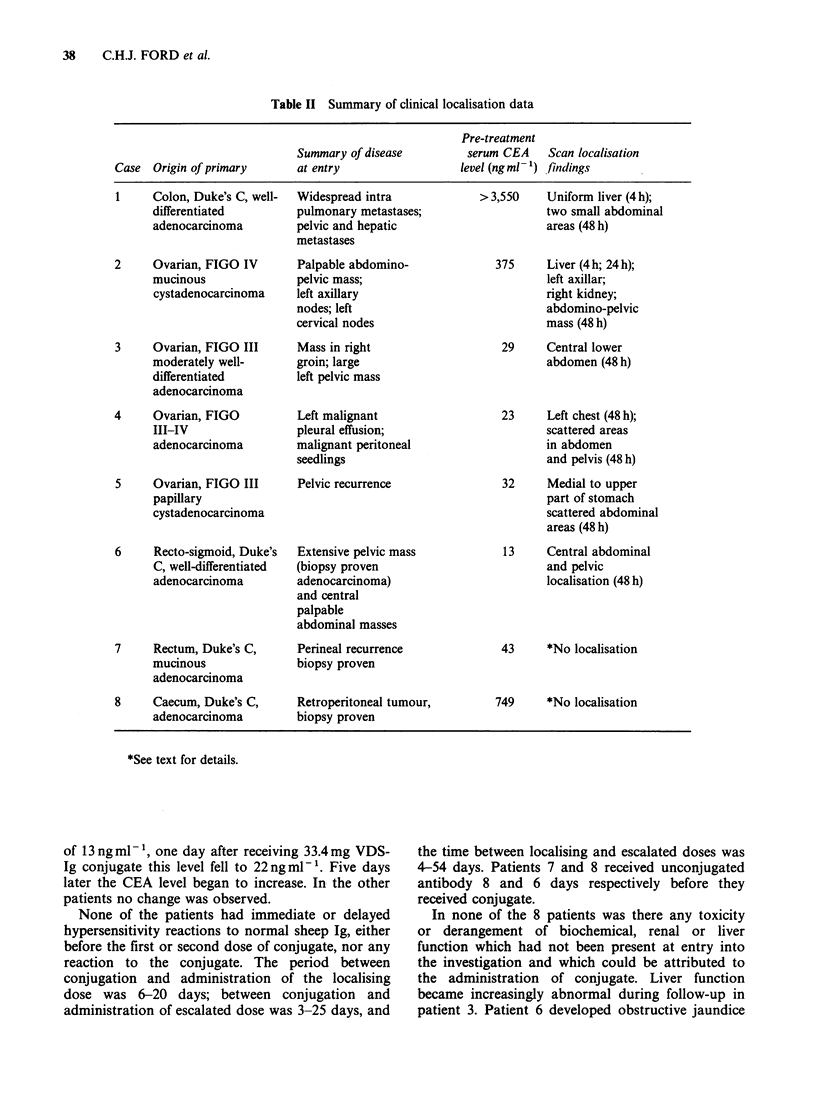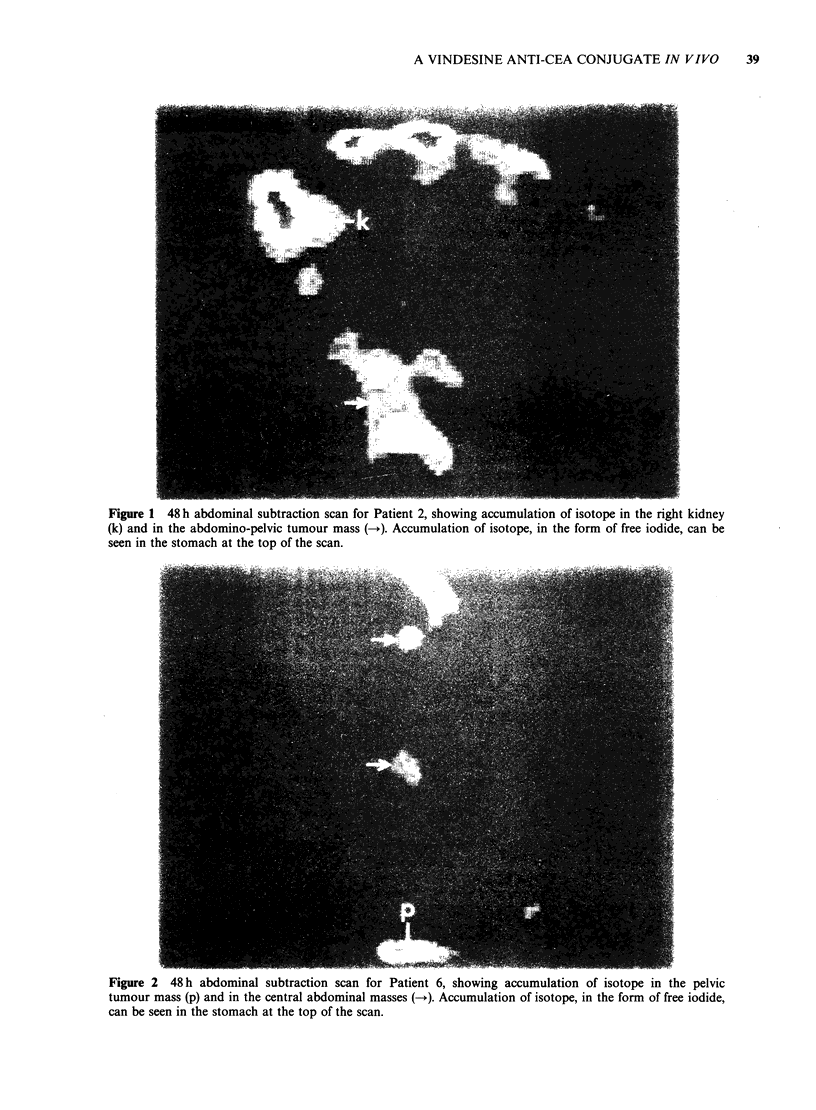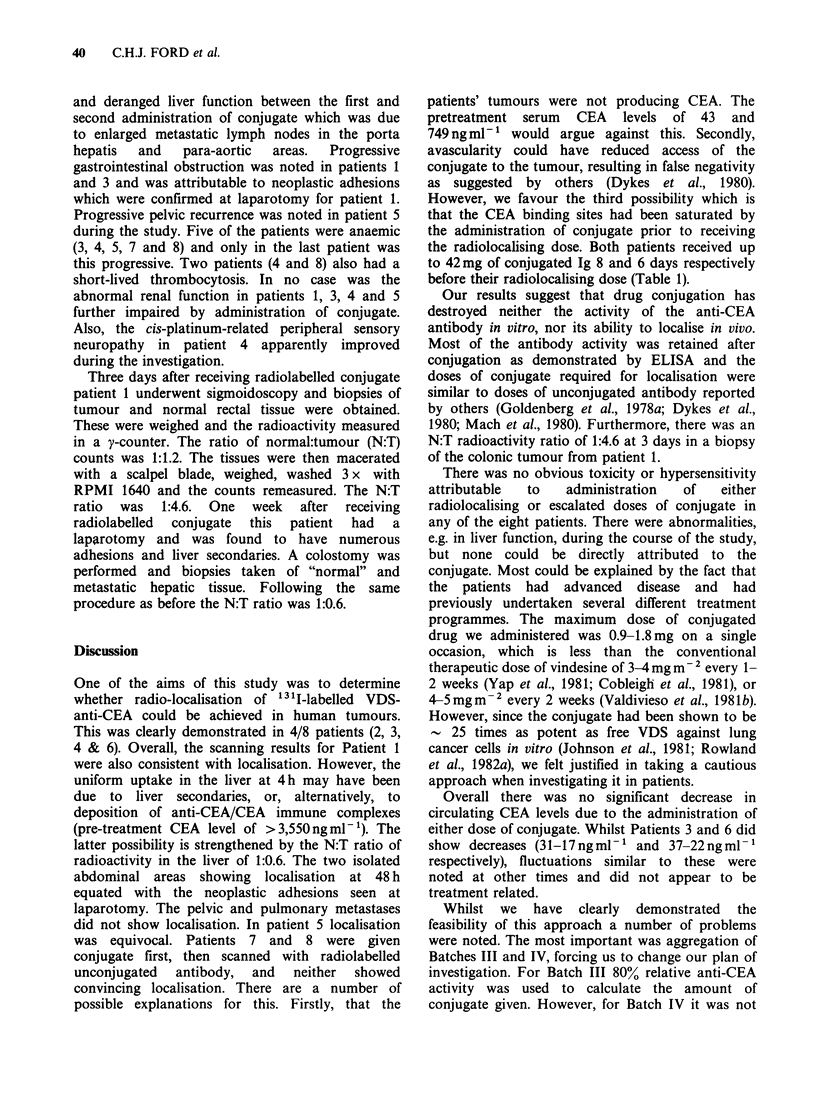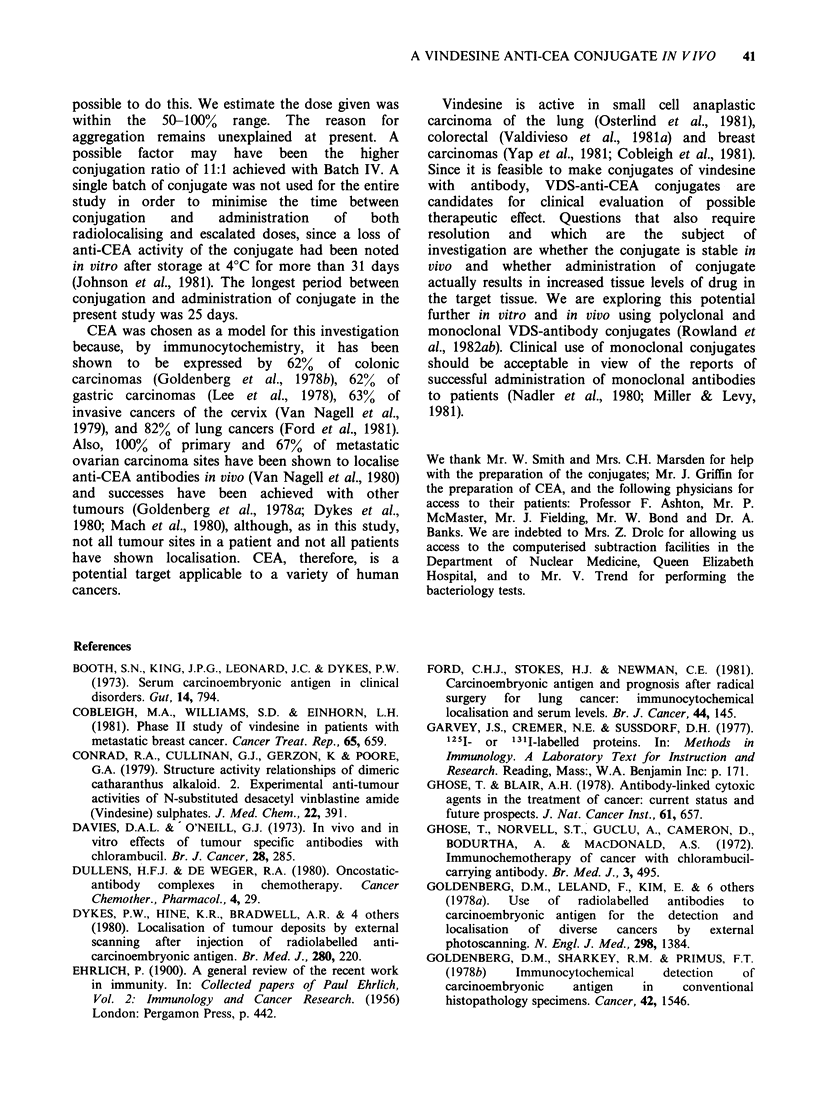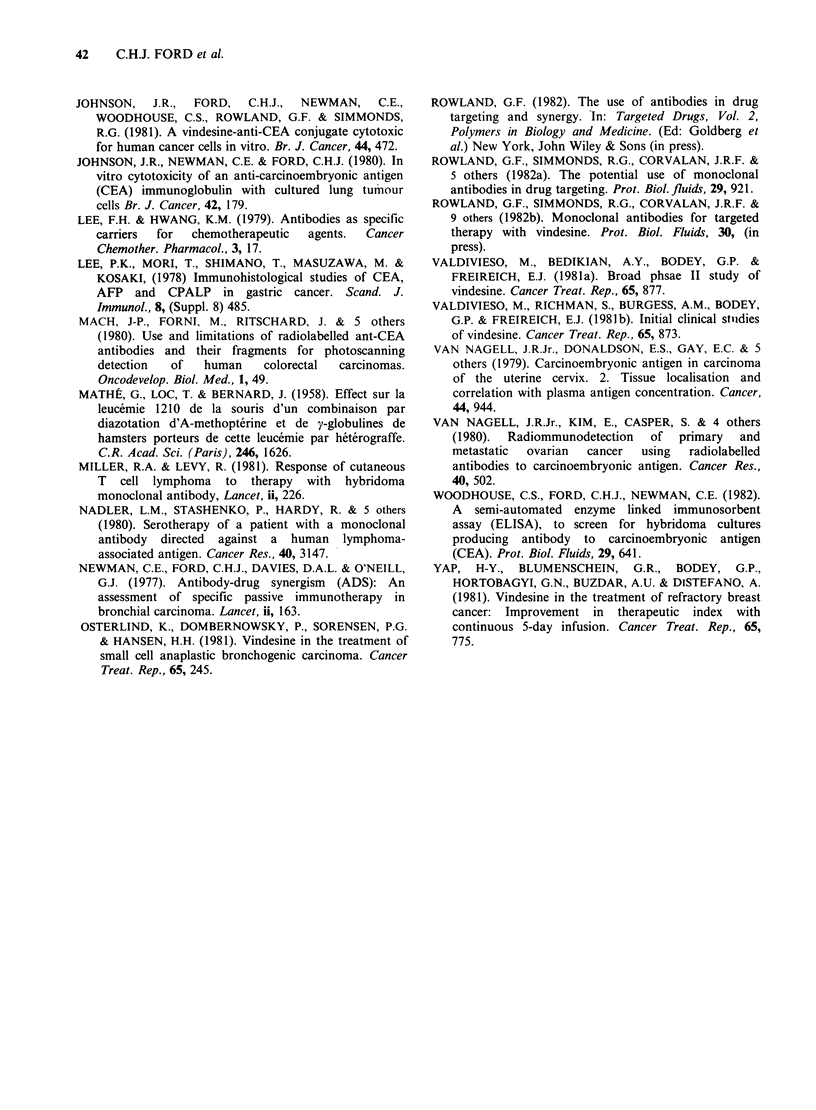# Localisation and toxicity study of a vindesine-anti-CEA conjugate in patients with advanced cancer.

**DOI:** 10.1038/bjc.1983.4

**Published:** 1983-01

**Authors:** C. H. Ford, C. E. Newman, J. R. Johnson, C. S. Woodhouse, T. A. Reeder, G. F. Rowland, R. G. Simmonds

## Abstract

**Images:**


*Br. J. Cancer* (1983), **47**, 035–042

### Localisation and toxicity study of a vindesine-anti-CEA conjugate in patients with advanced cancer

C.H.J. Ford[1]*, C.E. Newman[1]*, J.R. Johnson[1], C.S. Woodhouse[1], T.A. Reeder[2]‡, G.F. Rowland[3] & R.G. Simmonds[3]

[1]*Surgical Immunology Unit, Clinical Oncology,* [2]*the Dept. of Nuclear Medicine, Queen Elizabeth Hospital, Birmingham, and* [3]*the Lilly Research Centre, Erl Wood Manor, Windlesham, Surrey.*

**Summary** Safety of administration of a vindesine (VDS)-anti-CEA conjugate and its ability to localise after radiolabelling were investigated in patients with advanced metastatic carcinoma (4 colorectal and 4 ovarian). For imaging, patients received between 230 and 520 $\mu$g of $^{131}$I labelled antibody. In 5, localisation of conjugate was demonstrated, in another it was equivocal and in 2 patients, undetectable. For assessment of safety each patient also received a single dose of conjugate increasing from 1.2 to 42 mg antibody linked to 24 to 1800 $\mu$g VDS. The *in vitro* activity of the anti-CEA antibody and its ability to localise *in vivo* were preserved after conjugation. There was no obvious toxicity or hypersensitivity attributable to either the radiolocalisation or escalated doses of conjugate in any of the patients. The feasibility of the preparation and administration to patients of a vindesine-antibody conjugate has been demonstrated.

The concept of targeting drugs on to malignant cells proposed by Ehrlich (1900) offers a potential improvement over one of the major limitations of cancer chemotherapy, *viz.* the limited selectivity of drugs for cancer cells. The use of antibodies as carriers was tested experimentally in an L1210 leukaemia (Mathé *et al.*, 1958) and clinically in malignant melanoma (Ghose *et al.*, 1972).

Despite reports of some success with this approach it has not been widely adopted. This has been partly due to the inability to demonstrate tumour-specific antigens as targets in human tumours; however, in order to increase the therapeutic index of a drug, differential expression of the target by the tumour compared to normal tissue may be sufficient. The well characterised tumour-associated antigens, carcinoembryonic antigen (CEA) and alpha fetoprotein (AFP), offer such potential targets in man. Another reason for caution with this approach has been scepticism about the *in vivo* stability of the drug and antibody conjugate. It was demonstrated in the mouse EL4 lymphoma model, that drug and antibody were interactive and more effective than a conjugate of the two (Davies & O'Neill, 1973). This led to a pilot study of drug and antibody interaction in patients with resected bronchogenic carcinoma (Newman *et al.*, 1977) and to studies of a variety of drug and antibody conjugates and intermediate carriers (Ghose & Blair, 1978; Lee & Hwang, 1979; Dullens & De Weger, 1980, and Rowland, 1982).

The demonstration of enhanced toxicity of vincristine for a CEA-secreting lung cancer cell line in the presence of anti-CEA-immunoglobulin (Ig) (Johnson *et al.*, 1980) encouraged us to investigate direct conjugation of vinca alkaloids to antibody. The toxicity of vindesine for a CEA-secreting cell line was found to be greatly increased when conjugated to an anti-CEA-Ig (Johnson *et al.*, 1981). The aims of the present study were to investigate the ability to localise and safety of administration of this conjugate in patients with advanced metastatic adenocarcinomas refractory to established forms of treatment.

### Materials and methods

### *Patients*

Eight patients with advanced metastatic carcinoma refractory to previous treatment were entered into this study; all gave informed consent. Patients selected had tumour types likely to express CEA and to localise anti-CEA-antibodies (Goldenberg *et al.*, 1978*a*; Dykes *et al.*, 1980; Van Nagell *et al.*, 1980). Four had disseminated ovarian carcinomas and 4 had disseminated colorectal carcinomas. Before injection of radio-labelled antibody, patients were tested for immediate and delayed hypersensitivity to sheep Ig (0.1 ml of 1 mg ml$^{-1}$). When there was a delay of more than a week between the first dose of conjugate and the next dose sensitivity testing was repeated. To block

*Present address: Oncology Research, Memorial University and Newfoundland Cancer Clinic, Health Sciences Centre, St. John's, Newfoundland, Canada A1B 3V6.

‡Present address: Medical Physics Department, North Tees General Hospital, Stockton-on-Tees, Cleveland.

Received 29 June 1982; Accepted 23 September 1982.

0007-0920/83/010035–08 $01.00

© The Macmillan Press Ltd., 1983

thyroid uptake of $^{131}I$, potassium iodide tablets ($180 mg day^{-1}$) were given, beginning 1–3 days before administration of the iodinated conjugate/antibody and continuing for 8–12 days thereafter.

Patients were admitted to hospital 1–3 days prior to the study for complete physical examination, baseline laboratory investigations and hypersensitivity testing. Venous blood was sampled for full blood count, urea and electrolytes, biochemical profile and GTs. Twenty-four hour creatinine clearances were also performed. Temperature, blood pressure and pulse were recorded half-hourly during and for 4–6 h immediately after infusion of conjugate and every 4 h thereafter. Subjective toxicity was monitored daily by the attending physician (JRJ) and routine follow-up investigations performed every 1–2 days for the first 10–14 days. Particular attention was paid to evidence of hypersensitivity reactions and neurological status on clinical examination. Patients were then followed up in the clinic at weekly intervals for a minimum of 1 month, and in most cases 2–3 months.

*Antibody*

Immunoglobulin (Ig) was prepared from sheep anti-CEA serum by ammonium sulphate precipitation and was provided by Dr. A.R. Bradwell, Immunodiagnostics Research Laboratory, University of Birmingham, U.K. The antibody had been absorbed with normal liver, colon, lung and spleen. In fused rocket immunoelectrophoresis it did not recognise the CEA cross-reacting determinants shared with the non-specific cross-reacting antigen (NCA). Localisation of this antibody to human gastrointestinal tumour deposits has been reported (Dykes *et al.*, 1980). In our hands it localised on sections of a formalin-fixed CEA-secreting colonic carcinoma in an indirect immunoperoxidase test at a titre of 1/80,000. However, in the immunocytochemical tests there was still residual antibody activity to shared NCA determinants as demonstrated by staining of chronic myeloid leukaemia cells and splenic myeloid cells (Ford *et al.*, 1981).

*Conjugate*

Vindesine (VDS)-anti-CEA Ig conjugates were prepared at Lilly Research Centre Ltd. from desacetylvincaleucoblastine acid hydrazide under aseptic conditions by a modification of the procedure described for vindesine—BSA (Conrad *et al.*, 1979) and purified by gel filtration. Four batches were prepared with initial conjugation ratios of 4.1, 5.4, 4.3 and 11 moles vindesine per mole IgG. An iodinated aliquot of Batch I was used to scan patients 1 and 2. Iodinated Batch II was used to scan patients 3–6. Patients 7 and 8 were scanned with an iodinated aliquot of the sheep anti-CEA Ig used to prepare Batch IV.

For assessment of safety, patients received doses of 1.2–42 mg conjugate, containing 24–1800 $\mu$g VDS, injected i.v. in 100 ml of 1% human serum albumin in saline (HSA-saline) over a 30–60 min period. All conjugates were 0.22 $\mu$m filtered before dilution in sterile, HSA-saline.

*Radiolabelling of conjugate/antibody*

Aliquots of batches of conjugate (or antibody) were iodinated with $^{131}I$ using a modified chloramine-T method (Garvey *et al.*, 1977). Free iodine was removed on a Sephadex G-25 column which was eluted with HSA-saline. Fractions containing the protein peak as determined by $\gamma$-counting were pooled and sterile filtered through a 0.22 $\mu$m filter. Radiolabelled conjugate (or antibody) was injected i.v. in 100 ml of sterile HSA-saline over a 30–60 min period.

All solutions were tested for pyrogenicity and sterility. Iodinated conjugates were tested at $6 \mu g\, Ig\, kg^{-1}$ body weight in rabbits and uniodinated conjugates were tested from $45–198 \mu g\, Ig\, kg^{-1}$ body weight depending on the dose to be administered to patients. All batches were negative.

*Photoscanning*

Between 230–520 $\mu$g Ig conjugate (6–14 $\mu$g of VDS) containing 541–1014 $\mu$Ci protein bound $^{131}I$ was administered to patients 1–6. Patients 7 and 8 each received 471 $\mu$g of unconjugated Ig containing 1056 $\mu$Ci $^{131}I$.

Each patient received i.v. $^{99m}Tc$-pertechnetate (500 $\mu$Ci) 30 min before each scan, and $^{99m}Tc$-labelled human serum albumin (500 $\mu$Ci) 5 min before each scan. These distribute similarly to free iodide and radiolabelled antibody respectively in the blood pool. Images of the chest and abdomen were obtained with a gamma camera (Searle LFOV with medium energy collimator) initially at 4, 24 and 48 h after injection of iodinated material. The 4 h scans were discontinued for patients 3–8. The camera was linked to a DEC PDP11/40 computer with a dual isotope facility and a colour scale visual display unit. The data were stored and displayed in a $64 \times 64$ matrix. After normalising over the cardiac area, subtraction of the technetium component from the iodine component was performed to visualise areas of selective uptake of conjugate.

*Measurement of anti-CEA activity*

A modified enzyme-linked immunosorbent assay

(ELISA) (Woodhouse *et al.*, 1982) was used to measure anti-CEA activity of antibody, conjugate and iodinated conjugate. Briefly, disposable cuvettes were coated with purified CEA, washed, blocked by incubation with BSA, washed and the test sample added, before incubation at 35°C for 3 h. Following a further washing, rabbit anti-sheep IgG horseradish peroxidase conjugate (Nordic, U.K.) was added. The cuvettes were incubated at 35°C for 3 h, washed and then ABTS (2.2-azino-di-(3-ethylbenzthiazoline sulphonic acid) (Sigma)) was added. Absorbance at 405 nm was measured using a Gilford PR-50 processor-reader.

*Measurement of serum CEA levels*

CEA measurements were performed by Dr. P. Gosling, East Birmingham Hospital, using a modified double antibody technique (Booth *et al.*, 1973). Serum samples were perchloric acid-extracted before assay.

### Results

The dosages of radiolabelled conjugate/antibody and uniodinated conjugate received by each patient are given in Table I.

Details of the patients in this study and the scanning results are summarised in Table II. Patients 1–6 received radiolabelled conjugate, followed within 4–54 days by unlabelled conjugate. Localisation of radioactivity which equated with clinically detectable disease was seen in patients 1, 2, 3, 4 & 6. The localisation picture for patient 5 was equivocal. Figures 1 and 2 illustrate localisation images. Figure 1 is the 48 h subtraction scan for patient 2 who had a large abdomino-pelvic mass which was confirmed on CT scans. Localisation of isotope occurred in the mass and in the right kidney. Subsequent investigation of this patient by intravenous pyelography indicated a right hydronephrosis due to compression of the right ureter by the tumour. Impaired excretion apparently resulted in an accumulation of isotope in the right kidney.

Figure 2 is the 48 h subtraction scan for patient 6 who had an extensive pelvic tumour mass and central palpable abdominal masses. Ultrasound examination confirmed the presence of enlarged para-aortic and coeliac axis nodes. Localisation of isotope coinciding with these, and the pelvic mass at the primary site, can be seen in Figure 2.

For practical reasons, the last 2 patients received uniodinated conjugate and then radiolabelled unconjugated antibody. There was no evidence of localisation in these patients (see **Discussion**).

The relative anti-CEA activities of the non-iodinated conjugates in ELISA compared to the original antibody, when tested within 14 days of conjugation, were: Batch I, 98%; Batch II, 100%; Batch III, 80%. In the case of Batch IV it was not possible to obtain a relative anti-CEA value. After iodination the values were: Batch I, 48%; Batch II, 72%. Batches III and IV were not iodinated and the radiolabelled antibody used for patients 7 and 8 had 70% activity.

With the exception of patient 6, all had raised ($>15\,ng\,ml^{-1}$) pretreatment serum CEA levels (Table 2). We noted no significant decline in CEA levels in any of the patients after the radiolocalising dose. One day after administration of 11.06 mg VDS-Ig conjugate to patient 3, there was a fall from 31–17 $ng\,ml^{-1}$ which was sustained for 3 days. Similarly, for patient 6, who developed a raised CEA level of 37 $ng\,ml^{-1}$ from a pre-treatment value

**Table I** Summary of dosages

| | *Iodinated antibody* | | | *Non-iodinated antibody* | |
|---|---|---|---|---|---|
| *Patient* | VDS µg | Ig µg | $^{131}I$* µCi | VDS µg | Ig mg |
| 1 | 6 | 300 | 996 | 24.5 | 1.2 |
| 2 | 6 | 300 | 541 | 30.3 | 1.5 |
| 3 | 6.25 | 230 | 1014 | 300.8 | 11.06 |
| 4 | 6.5 | 240 | 581 | 300.8 | 11.06 |
| 5 | 14.1 | 520 | 633 | 722 | 33.4 |
| 6 | 14.1 | 520 | 633 | 722 | 33.4 |
| 7 | — | 471 | 1056 | 924.4– | 20.9– |
| 8 | — | 471 | 1056 | 1849‡ | 41.8‡ |

*protein-associated radioactivity.
‡dose given was within this estimated range—see **Discussion**.

**Table II** Summary of clinical localisation data

| *Case* | *Origin of primary* | *Summary of disease at entry* | *Pre-treatment serum CEA level* ($ng\,ml^{-1}$) | *Scan localisation findings* |
|---|---|---|---|---|
| 1 | Colon, Duke's C, well-differentiated adenocarcinoma | Widespread intra pulmonary metastases; pelvic and hepatic metastases | >3,550 | Uniform liver (4 h); two small abdominal areas (48 h) |
| 2 | Ovarian, FIGO IV mucinous cystadenocarcinoma | Palpable abdomino-pelvic mass; left axillary nodes; left cervical nodes | 375 | Liver (4 h; 24 h); left axillar; right kidney; abdomino-pelvic mass (48 h) |
| 3 | Ovarian, FIGO III moderately well-differentiated adenocarcinoma | Mass in right groin; large left pelvic mass | 29 | Central lower abdomen (48 h) |
| 4 | Ovarian, FIGO III–IV adenocarcinoma | Left malignant pleural effusion; malignant peritoneal seedlings | 23 | Left chest (48 h); scattered areas in abdomen and pelvis (48 h) |
| 5 | Ovarian, FIGO III papillary cystadenocarcinoma | Pelvic recurrence | 32 | Medial to upper part of stomach scattered abdominal areas (48 h) |
| 6 | Recto-sigmoid, Duke's C, well-differentiated adenocarcinoma | Extensive pelvic mass (biopsy proven adenocarcinoma) and central palpable abdominal masses | 13 | Central abdominal and pelvic localisation (48 h) |
| 7 | Rectum, Duke's C, mucinous adenocarcinoma | Perineal recurrence biopsy proven | 43 | *No localisation |
| 8 | Caecum, Duke's C, adenocarcinoma | Retroperitoneal tumour, biopsy proven | 749 | *No localisation |

*See text for details.

of $13\,ng\,ml^{-1}$, one day after receiving 33.4 mg VDS-Ig conjugate this level fell to $22\,ng\,ml^{-1}$. Five days later the CEA level began to increase. In the other patients no change was observed.

None of the patients had immediate or delayed hypersensitivity reactions to normal sheep Ig, either before the first or second dose of conjugate, nor any reaction to the conjugate. The period between conjugation and administration of the localising dose was 6–20 days; between conjugation and administration of escalated dose was 3–25 days, and the time between localising and escalated doses was 4–54 days. Patients 7 and 8 received unconjugated antibody 8 and 6 days respectively before they received conjugate.

In none of the 8 patients was there any toxicity or derangement of biochemical, renal or liver function which had not been present at entry into the investigation and which could be attributed to the administration of conjugate. Liver function became increasingly abnormal during follow-up in patient 3. Patient 6 developed obstructive jaundice

**Figure 1** 48 h abdominal subtraction scan for Patient 2, showing accumulation of isotope in the right kidney (k) and in the abdomino-pelvic tumour mass (→). Accumulation of isotope, in the form of free iodide, can be seen in the stomach at the top of the scan.

**Figure 2** 48 h abdominal subtraction scan for Patient 6, showing accumulation of isotope in the pelvic tumour mass (p) and in the central abdominal masses (→). Accumulation of isotope, in the form of free iodide, can be seen in the stomach at the top of the scan.

and deranged liver function between the first and second administration of conjugate which was due to enlarged metastatic lymph nodes in the porta hepatis and para-aortic areas. Progressive gastrointestinal obstruction was noted in patients 1 and 3 and was attributable to neoplastic adhesions which were confirmed at laparotomy for patient 1. Progressive pelvic recurrence was noted in patient 5 during the study. Five of the patients were anaemic (3, 4, 5, 7 and 8) and only in the last patient was this progressive. Two patients (4 and 8) also had a short-lived thrombocytosis. In no case was the abnormal renal function in patients 1, 3, 4 and 5 further impaired by administration of conjugate. Also, the *cis*-platinum-related peripheral sensory neuropathy in patient 4 apparently improved during the investigation.

Three days after receiving radiolabelled conjugate patient 1 underwent sigmoidoscopy and biopsies of tumour and normal rectal tissue were obtained. These were weighed and the radioactivity measured in a $\gamma$-counter. The ratio of normal:tumour (N:T) counts was 1:1.2. The tissues were then macerated with a scalpel blade, weighed, washed 3 × with RPMI 1640 and the counts remeasured. The N:T ratio was 1:4.6. One week after receiving radiolabelled conjugate this patient had a laparotomy and was found to have numerous adhesions and liver secondaries. A colostomy was performed and biopsies taken of "normal" and metastatic hepatic tissue. Following the same procedure as before the N:T ratio was 1:0.6.

### Discussion

One of the aims of this study was to determine whether radio-localisation of $^{131}$I-labelled VDS-anti-CEA could be achieved in human tumours. This was clearly demonstrated in 4/8 patients (2, 3, 4 & 6). Overall, the scanning results for Patient 1 were also consistent with localisation. However, the uniform uptake in the liver at 4 h may have been due to liver secondaries, or, alternatively, to deposition of anti-CEA/CEA immune complexes (pre-treatment CEA level of $>3,550\,ng\,ml^{-1}$). The latter possibility is strengthened by the N:T ratio of radioactivity in the liver of 1:0.6. The two isolated abdominal areas showing localisation at 48 h equated with the neoplastic adhesions seen at laparotomy. The pelvic and pulmonary metastases did not show localisation. In patient 5 localisation was equivocal. Patients 7 and 8 were given conjugate first, then scanned with radiolabelled unconjugated antibody, and neither showed convincing localisation. There are a number of possible explanations for this. Firstly, that the patients' tumours were not producing CEA. The pretreatment serum CEA levels of 43 and $749\,ng\,ml^{-1}$ would argue against this. Secondly, avascularity could have reduced access of the conjugate to the tumour, resulting in false negativity as suggested by others (Dykes *et al.*, 1980). However, we favour the third possibility which is that the CEA binding sites had been saturated by the administration of conjugate prior to receiving the radiolocalising dose. Both patients received up to 42 mg of conjugated Ig 8 and 6 days respectively before their radiolocalising dose (Table 1).

Our results suggest that drug conjugation has destroyed neither the activity of the anti-CEA antibody *in vitro*, nor its ability to localise *in vivo*. Most of the antibody activity was retained after conjugation as demonstrated by ELISA and the doses of conjugate required for localisation were similar to doses of unconjugated antibody reported by others (Goldenberg *et al.*, 1978*a*; Dykes *et al.*, 1980; Mach *et al.*, 1980). Furthermore, there was an N:T radioactivity ratio of 1:4.6 at 3 days in a biopsy of the colonic tumour from patient 1.

There was no obvious toxicity or hypersensitivity attributable to administration of either radiolocalising or escalated doses of conjugate in any of the eight patients. There were abnormalities, e.g. in liver function, during the course of the study, but none could be directly attributed to the conjugate. Most could be explained by the fact that the patients had advanced disease and had previously undertaken several different treatment programmes. The maximum dose of conjugated drug we administered was 0.9–1.8 mg on a single occasion, which is less than the conventional therapeutic dose of vindesine of $3–4\,mg\,m^{-2}$ every 1–2 weeks (Yap *et al.*, 1981; Cobleigh *et al.*, 1981), or $4–5\,mg\,m^{-2}$ every 2 weeks (Valdivieso *et al.*, 1981*b*). However, since the conjugate had been shown to be ~ 25 times as potent as free VDS against lung cancer cells *in vitro* (Johnson *et al.*, 1981; Rowland *et al.*, 1982*a*), we felt justified in taking a cautious approach when investigating it in patients.

Overall there was no significant decrease in circulating CEA levels due to the administration of either dose of conjugate. Whilst Patients 3 and 6 did show decreases ($31–17\,ng\,ml^{-1}$ and $37–22\,ng\,ml^{-1}$ respectively), fluctuations similar to these were noted at other times and did not appear to be treatment related.

Whilst we have clearly demonstrated the feasibility of this approach a number of problems were noted. The most important was aggregation of Batches III and IV, forcing us to change our plan of investigation. For Batch III 80% relative anti-CEA activity was used to calculate the amount of conjugate given. However, for Batch IV it was not possible to do this. We estimate the dose given was within the 50–100% range. The reason for aggregation remains unexplained at present. A possible factor may have been the higher conjugation ratio of 11:1 achieved with Batch IV. A single batch of conjugate was not used for the entire study in order to minimise the time between conjugation and administration of both radiolocalising and escalated doses, since a loss of anti-CEA activity of the conjugate had been noted *in vitro* after storage at 4°C for more than 31 days (Johnson *et al*., 1981). The longest period between conjugation and administration of conjugate in the present study was 25 days.

CEA was chosen as a model for this investigation because, by immunocytochemistry, it has been shown to be expressed by 62% of colonic carcinomas (Goldenberg *et al*., 1978*b*), 62% of gastric carcinomas (Lee *et al*., 1978), 63% of invasive cancers of the cervix (Van Nagell *et al*., 1979), and 82% of lung cancers (Ford *et al*., 1981). Also, 100% of primary and 67% of metastatic ovarian carcinoma sites have been shown to localise anti-CEA antibodies *in vivo* (Van Nagell *et al*., 1980) and successes have been achieved with other tumours (Goldenberg *et al*., 1978*a*; Dykes *et al*., 1980; Mach *et al*., 1980), although, as in this study, not all tumour sites in a patient and not all patients have shown localisation. CEA, therefore, is a potential target applicable to a variety of human cancers.

Vindesine is active in small cell anaplastic carcinoma of the lung (Osterlind *et al*., 1981), colorectal (Valdivieso *et al*., 1981*a*) and breast carcinomas (Yap *et al*., 1981; Cobleigh *et al*., 1981). Since it is feasible to make conjugates of vindesine with antibody, VDS-anti-CEA conjugates are candidates for clinical evaluation of possible therapeutic effect. Questions that also require resolution and which are the subject of investigation are whether the conjugate is stable *in vivo* and whether administration of conjugate actually results in increased tissue levels of drug in the target tissue. We are exploring this potential further *in vitro* and *in vivo* using polyclonal and monoclonal VDS-antibody conjugates (Rowland *et al*., 1982*ab*). Clinical use of monoclonal conjugates should be acceptable in view of the reports of successful administration of monoclonal antibodies to patients (Nadler *et al*., 1980; Miller & Levy, 1981).

We thank Mr. W. Smith and Mrs. C.H. Marsden for help with the preparation of the conjugates; Mr. J. Griffin for the preparation of CEA, and the following physicians for access to their patients: Professor F. Ashton, Mr. P. McMaster, Mr. J. Fielding, Mr. W. Bond and Dr. A. Banks. We are indebted to Mrs. Z. Drolc for allowing us access to the computerised subtraction facilities in the Department of Nuclear Medicine, Queen Elizabeth Hospital, and to Mr. V. Trend for performing the bacteriology tests.

### References

BOOTH, S.N., KING, J.P.G., LEONARD, J.C. & DYKES, P.W. (1973). Serum carcinoembryonic antigen in clinical disorders. *Gut*, **14**, 794.

COBLEIGH, M.A., WILLIAMS, S.D. & EINHORN, L.H. (1981). Phase II study of vindesine in patients with metastatic breast cancer. *Cancer Treat. Rep.*, **65**, 659.

CONRAD, R.A., CULLINAN, G.J., GERZON, K & POORE, G.A. (1979). Structure activity relationships of dimeric catharanthus alkaloid. 2. Experimental anti-tumour activities of N-substituted desacetyl vinblastine amide (Vindesine) sulphates. *J. Med. Chem.*, **22**, 391.

DAVIES, D.A.L. & O'NEILL, G.J. (1973). In vivo and in vitro effects of tumour specific antibodies with chlorambucil. *Br. J. Cancer*, **28**, 285.

DULLENS, H.F.J. & DE WEGER, R.A. (1980). Oncostatic-antibody complexes in chemotherapy. *Cancer Chemother., Pharmacol.*, **4**, 29.

DYKES, P.W., HINE, K.R., BRADWELL, A.R. & 4 others (1980). Localisation of tumour deposits by external scanning after injection of radiolabelled anti-carcinoembryonic antigen. *Br. Med. J.*, **280**, 220.

EHRLICH, P. (1900). A general review of the recent work in immunity. In: *Collected papers of Paul Ehrlich, Vol. 2: Immunology and Cancer Research*. (1956) London: Pergamon Press, p. 442.

FORD, C.H.J., STOKES, H.J. & NEWMAN, C.E. (1981). Carcinoembryonic antigen and prognosis after radical surgery for lung cancer: immunocytochemical localisation and serum levels. *Br. J. Cancer*, **44**, 145.

GARVEY, J.S., CREMER, N.E. & SUSSDORF, D.H. (1977). $^{125}$I- or $^{131}$I-labelled proteins. In: *Methods in Immunology. A Laboratory Text for Instruction and Research*. Reading, Mass:, W.A. Benjamin Inc: p. 171.

GHOSE, T. & BLAIR, A.H. (1978). Antibody-linked cytoxic agents in the treatment of cancer: current status and future prospects. *J. Nat. Cancer Inst.*, **61**, 657.

GHOSE, T., NORVELL, S.T., GUCLU, A., CAMERON, D., BODURTHA, A. & MACDONALD, A.S. (1972). Immunochemotherapy of cancer with chlorambucil-carrying antibody. *Br. Med. J.*, **3**, 495.

GOLDENBERG, D.M., LELAND, F., KIM, E. & 6 others (1978*a*). Use of radiolabelled antibodies to carcinoembryonic antigen for the detection and localisation of diverse cancers by external photoscanning. *N. Engl. J. Med.*, **298**, 1384.

GOLDENBERG, D.M., SHARKEY, R.M. & PRIMUS, F.T. (1978*b*) Immunocytochemical detection of carcinoembryonic antigen in conventional histopathology specimens. *Cancer*, **42**, 1546.

JOHNSON, J.R., FORD, C.H.J., NEWMAN, C.E., WOODHOUSE, C.S., ROWLAND, G.F. & SIMMONDS, R.G. (1981). A vindesine-anti-CEA conjugate cytotoxic for human cancer cells in vitro. *Br. J. Cancer*, **44**, 472.

JOHNSON, J.R., NEWMAN, C.E. & FORD, C.H.J. (1980). In vitro cytotoxicity of an anti-carcinoembryonic antigen (CEA) immunoglobulin with cultured lung tumour cells *Br. J. Cancer*, **42**, 179.

LEE, F.H. & HWANG, K.M. (1979). Antibodies as specific carriers for chemotherapeutic agents. *Cancer Chemother. Pharmacol.*, **3**, 17.

LEE, P.K., MORI, T., SHIMANO, T., MASUZAWA, M. & KOSAKI, (1978) Immunohistological studies of CEA, AFP and CPALP in gastric cancer. *Scand. J. Immunol.*, **8**, (Suppl. 8) 485.

MACH, J-P., FORNI, M., RITSCHARD, J. & 5 others (1980). Use and limitations of radiolabelled ant-CEA antibodies and their fragments for photoscanning detection of human colorectal carcinomas. *Oncodevelop. Biol. Med.*, **1**, 49.

MATHÉ, G., LOC, T. & BERNARD, J. (1958). Effect sur la leucémie 1210 de la souris d'un combinaison par diazotation d'A-methoptérine et de γ-globulines de hamsters porteurs de cette leucémie par hétérograffe. *C.R. Acad. Sci. (Paris)*, **246**, 1626.

MILLER, R.A. & LEVY, R. (1981). Response of cutaneous T cell lymphoma to therapy with hybridoma monoclonal antibody, *Lancet*, **ii**, 226.

NADLER, L.M., STASHENKO, P., HARDY, R. & 5 others (1980). Serotherapy of a patient with a monoclonal antibody directed against a human lymphoma-associated antigen. *Cancer Res.*, **40**, 3147.

NEWMAN, C.E., FORD, C.H.J., DAVIES, D.A.L. & O'NEILL, G.J. (1977). Antibody-drug synergism (ADS): An assessment of specific passive immunotherapy in bronchial carcinoma. *Lancet*, **ii**, 163.

OSTERLIND, K., DOMBERNOWSKY, P., SORENSEN, P.G. & HANSEN, H.H. (1981). Vindesine in the treatment of small cell anaplastic bronchogenic carcinoma. *Cancer Treat. Rep.*, **65**, 245.

ROWLAND, G.F. (1982). The use of antibodies in drug targeting and synergy. In: *Targeted Drugs, Vol. 2, Polymers in Biology and Medicine*. (Ed: Goldberg *et al.*) New York, John Wiley & Sons (in press).

ROWLAND, G.F., SIMMONDS, R.G., CORVALAN, J.R.F. & 5 others (1982a). The potential use of monoclonal antibodies in drug targeting. *Prot. Biol. fluids*, **29**, 921.

ROWLAND, G.F., SIMMONDS, R.G., CORVALAN, J.R.F. & 9 others (1982b). Monoclonal antibodies for targeted therapy with vindesine. *Prot. Biol. Fluids*, **30**, (in press).

VALDIVIESO, M., BEDIKIAN, A.Y., BODEY, G.P. & FREIREICH, E.J. (1981a). Broad phsae II study of vindesine. *Cancer Treat. Rep.*, **65**, 877.

VALDIVIESO, M., RICHMAN, S., BURGESS, A.M., BODEY, G.P. & FREIREICH, E.J. (1981b). Initial clinical studies of vindesine. *Cancer Treat. Rep.*, **65**, 873.

VAN NAGELL, J.R.Jr., DONALDSON, E.S., GAY, E.C. & 5 others (1979). Carcinoembryonic antigen in carcinoma of the uterine cervix. 2. Tissue localisation and correlation with plasma antigen concentration. *Cancer*, **44**, 944.

VAN NAGELL, J.R.Jr., KIM, E., CASPER, S. & 4 others (1980). Radiommunodetection of primary and metastatic ovarian cancer using radiolabelled antibodies to carcinoembryonic antigen. *Cancer Res.*, **40**, 502.

WOODHOUSE, C.S., FORD, C.H.J., NEWMAN, C.E. (1982). A semi-automated enzyme linked immunosorbent assay (ELISA), to screen for hybridoma cultures producing antibody to carcinoembryonic antigen (CEA). *Prot. Biol. Fluids*, **29**, 641.

YAP, H-Y., BLUMENSCHEIN, G.R., BODEY, G.P., HORTOBAGYI, G.N., BUZDAR, A.U. & DISTEFANO, A. (1981). Vindesine in the treatment of refractory breast cancer: Improvement in therapeutic index with continuous 5-day infusion. *Cancer Treat. Rep.*, **65**, 775.